# Seeking virtual social support through blogging: A content analysis of published blog posts written by people with chronic pain

**DOI:** 10.1177/2055207618772669

**Published:** 2018-05-16

**Authors:** Samuel Tsai, Emma Crawford, Jenny Strong

**Affiliations:** School of Health and Rehabilitation Sciences, The University of Queensland, Australia

**Keywords:** Chronic pain, blogging, virtual social support, social support, pain, digital media, social media

## Abstract

**Objective:**

People with chronic pain often have limited avenues for social support. Social isolation often develops as their abilities to engage in daily social and vocational activities decrease. With recent advancements in technology and increasing use of social media, virtual platforms such as blogging may provide opportunities for social support. This study analyzed published blog posts of people with chronic pain to investigate how social support occurs through blogging for chronic pain blog users and the nature of such online interactions.

**Methods:**

A total of 810 blog posts published from January 2014 to December 2015 on 44 publicly accessible chronic pain blogs were collected and analyzed through qualitative phenomenological thematic analysis.

**Results:**

The Virtual Online Support Sequence (VOSS) was identified through the exchange of online comments; this sequence defines the process by which virtual social support can be established through the process of chronic pain blogging. Three subthemes were also identified in relation to social support in the online blogging environment: (a) the virtual community of pain blogging; (b) establishing social support through the VOSS; and (c) recounting everyday experiences related to pain.

**Conclusions:**

These findings suggest that blogging can be useful in seeking, receiving and providing social support for people with chronic pain. Understanding this mechanism behind establishing virtual social support may potentially encourage people with chronic pain to pursue additional support online if they have limited face-to-face opportunities.

## Introduction

Online social media has created opportunities for people with chronic pain to find and communicate with others with similar health problems. These online platforms provide an affordable and convenient means of direct, community and mass communication.^1^ Blogging, the process of regular online journaling,^2^ can allow people to create, document and share their experiences of living with chronic pain.^3^

Family and friends may lack a nuanced understanding of the experience of chronic pain and its debilitating impacts.^4,5^ Such a situation may create experiences of social exclusion and isolation for people with chronic pain as the ability to access social support in forms of emotional, instrumental and information resources can be limited in times of need.^6^ Social support is beneficial for people with chronic pain because it fosters positive pain management strategy use and reduces feelings of isolation.^7^ Thus social media platforms, such as blogs, can be important spaces for social connection and support for people with chronic pain who may lack social support in other areas of their lives.

Pain is considered chronic when it persists longer than the anticipated tissue healing time and when it serves no useful role in recovery.^8^ Chronic pain may develop from long-term conditions such as arthritis, cancer and diabetes or from damage to the central or peripheral nervous systems.^9^ Chronic pain is a common experience within the community, despite age, gender, culture or geography,^10^ and affects 1 in 10 adults in the general population.^11^ While access to pain management is recognized as a fundamental human right (as declared in the Declaration of Montréal),^12^ chronic pain continues to burden healthcare systems significantly with its variable causes, presentation, low recovery rates and poor response to treatment options.^10,11^ As such, the experience of chronic pain can impact on an individual's capacity and willingness to participate in everyday, social and vocational activities.^13^

When documenting personal narratives through online journals such as blogs, experiential history is central.^14^ These stories provide insight into an individual's everyday life, capture their experiences over time and can create a community of support.^14,15^ Bloggers often disclose honest, personal narratives when they publish content online aimed towards readers who can interact via feedback through comments.^16,17^ Research has also posited blogging as a useful communication medium of virtual social support for people with a variety of health conditions.^13,18–21^ As routine daily activities such as work are known to provide a sense of social and emotional support for people,^22^ regular blogging is potentially useful for people with chronic pain as it provides a conduit to enable them to connect with others who understand and share their experiences of pain,^7,22^ possibly encouraging increased participation in personally meaningful life activities, positive pain management experiences, and social connectedness.

While there is literature indicating that blogging can enable people with a range of illnesses to communicate and exchange resources and information,^20^ there is currently limited research investigating the way in which people with chronic pain use blogging. One survey conducted with individuals with chronic pain has provided preliminary insights into the perceived psychosocial and health benefits of blogging.^13^ Two hundred and thirty participants indicated that blogging was beneficial as it provided an expressive outlet, allowed opportunities for them to reframe their chronic pain experiences and provided opportunities for feedback and support.^13^ These findings are similar to a later survey where people with chronic pain reported that blogging increased support from others, enjoyment of life and participation in social activities.^23^ However, neither study indicated the mechanism behind how blogging positively influenced people with chronic pain nor how virtual social support occurred.

Current understanding of how blogging provides virtual social support is drawn from a small number of qualitative studies about people with a range of health conditions such as cancer, diabetes, depression and heart disease. These studies have indicated that opportunities for reflection, individual expression, emotional catharsis and a sense of relatedness with other blog users can be obtained by sharing individual illness experiences, perspectives and knowledge.^2,21,23^ Simply reading similar experiences can be motivating and help to reframe illness experiences.^21^ However, it is through commenting on blog posts that bloggers and readers are able to enter into interactive social encounters, approximating the cyclic support interactions of encouragement and emotional support that are usually gained through face-to-face interactions.^20,21^ What these cyclic support interactions entail is yet to be examined.

Overall, social media platforms act as convenient mediums to access social support and have been recognized to provide psychosocial benefits in reducing loneliness and anxiety and helping with the acceptance of pain.^13,16,19,21,24–29^ More specifically, blogging can reduce a sense of isolation among bloggers and blog readers through mutual sharing of individual illness experiences.^21,23^ For people with chronic pain, blogging has also been found to promote health-related actions and encourage the use of adaptive coping strategies.^13,21^ However, little is known about the processes through which online interactions can provide social support specifically for people with chronic pain. This study examines the experiences of chronic pain blog users to gain an understanding of how social support occurs through blogging and the nature, content and style of such online interactions.

## Methods

Qualitative inquiry can facilitate new areas of research by providing descriptive insights into the nature or meaning of everyday experiences.^30^ Qualitative phenomenological thematic analysis aims to describe the meaning, structure and essence of a lived experience and was used in this study to understand the experiences of chronic pain blog users.^30^

While interviews are commonly used as a data source for phenomenological research,^30^ digital media content, such as blogs, is a valid source of data for understanding experiences of chronic pain. This is especially true when it is used as a form of online journaling or expression of individual thoughts, feelings and experiences.^31^ Individual blog posts provide honest and detailed descriptions of chronic pain experiences and, when examined together, they provide a holistic insight into life with chronic pain.^16^ Therefore, by observing and analyzing content from blog posts and interactions between blog users, it is possible to gain a thorough understanding into the experiences of chronic pain blog users without conducting interviews.

Due to blog-hosting websites of Blogger, Tumblr, WordPress, Weebly and StudioPress stating that content published on their websites is publically available and the freely accessible nature of information on public blog domains, consent was assumed in this study.^32,33^ This method of collecting data was approved by The University of Queensland's Behavioural and Social Sciences Ethical Review Committee (clearance #2013001316). To lower the traceability of quotes on search engines, names of blogs and blog users have been omitted and short segments from blog posts were used as quotes.^32,33^

### Sample

Forty-four publicly accessible blogs with entries focused on individual pain experiences constituted the data for this study. To examine these experiences, blogs were authored by people who reported having a diagnosis of chronic pain or were experiencing chronic pain as a symptom of a chronic illness within their blogs. Blogs were selected if there were entries published between January 2014 and December 2015, with at least one blog post in December 2015. Blogs were included in this study regardless of the country from which authors were documenting their experiences from. Blogs that featured a mixed focus of both pain experiences and another topic, such as beauty or fashion, were not included. Furthermore, only blogs from authors aged 18 years or over in January 2014 were included.

A combination of search terms was used to retrieve this intensity sample. The following Boolean search string indicates terms used to identify blogs: (“chronic pain”OR“persistent pain”)AND(“blogger”OR“blogs”OR“tumblr”OR“blogspot”OR“wordpress”)AND(“2014”OR“2015”). Searches were conducted using the internet search engine Google and within the blog-hosting websites of WordPress and Tumblr. Snowball sampling by following blog text recommending other chronic pain blogs was also used.^33^

### Data collection

While 45 blogs met the inclusion criteria, data for only 44 blogs could be collected and analyzed due to broken links within one blog site. For each blog, data were collected manually from blog posts and gathered into a common document. For each blog post, the elements collected for analysis included the blog post title, blog post text and blog post comments. Table 1 provides an example of each blog post element. Other data collected from blogs included blog author gender, occupation, condition/s and country from which they were blogging; however, this demographical information was not analyzed further in this study.

**Table 1.** Description and example of blog post elements.

Blog post element	Description	Example
Title	Titles provided an introduction to the blog post content.	“Doctor Vs Patient”
Text	Text, in the form of paragraphs, formed majority of blog posts providing information about a blogger's experience of chronic pain.	“The duties of a doctor are to take care of his/her patient, protect and promote the public and the patients’ health … A doctor's duty is not to criticize or make their patients feel like an idiot or guilty.”
Comment	Comments were written and published by readers in response to the author's blog post. Comments appeared at the end of blog posts. Comments could be made anonymously. Comment exchanges occurred when a series of replies between different commenters occurred. An example of a comment exchange between a reader and blogger is provided in the next column.	“Anyway, I’m so sorry to hear of your frustrations … I am very frustrated today too, but in a different way (I just wrote a post this morning too). So grateful to be doing so well, but still having major hand issues despite making so much progress.”
		“Thank you for your reply … I’m sorry you're frustrated with your hands and I hope it passes soon. You're a warrior and I hope you realize meltdowns are totally ok and totally rad to do.”

There was a total of 9719 blog posts within the 24-month period. To capture and critically understand the progression of each author's chronic pain experience over time, only the first blog post of every month from each blog over the 24-month period was used for analysis. This resulted in a sample of 810 blog posts from 44 blogs about chronic pain experiences.

This study focused on the experiences of blog authors. Therefore, only original blog posts that were written and published by blog authors, and their associated comments, were analyzed. This included people with chronic pain and their guest bloggers. Guest blogging occurred in some instances when blog authors invited their partners to describe their experiences as caregivers. Two guest posts were included in analysis as they were original entries providing information about living with pain. Content that was re-blogged, a mechanism allowing blog authors to repost content created by someone else, was not analyzed. Seventy-five blog posts out of the 810 posts sampled did not contain content about experiences of pain and were excluded from analysis. Therefore, a total of 735 blog posts was included in the analysis.

### Data analysis

Content analysis was conducted through paragraph-by-paragraph coding of blog posts. Codes were assigned inductively and involved reading paragraphs and creating codes when concepts were identified.^34^ Constant comparison between data and codes allowed for paragraphs assigned the same code to be compared and adjusted and for new codes to be created to distinguish between similarities and differences of paragraph content.^33^ One investigator conducted the initial coding. A second researcher reviewed codes and compared them to the data. Some revision of codes occurred and some additional codes were added until complete consensus between researchers regarding codes was achieved. Memos and diagrams describing identified concepts were used adjunctively to develop understanding of patterns from the data.^35^

Similar codes were grouped together, leading to 18 different categories being identified. For example, the codes “masking pain from others”, “perceived ideas of pain”, “lack of knowledge about pain” and “insensitivity from others” were grouped together under the category “difficulty communicating with others about pain”. These 18 categories were then reviewed and ordered into four different levels of themes with three overarching first order themes: (a) social support in the online blogging environment; (b) experiences of life with pain; and (c) sharing information with readers. Supplementary Table 1 outlines the overarching themes, categories and codes identified in data anlaysis. This paper reports on the overarching theme that describes how social support occurs in online blogging environments.

## Results

Analysis identified three subthemes related to social support in the online blogging environment: (a) the virtual community of pain blogging; (b) establishing social support through the Virtual Online Support Sequence (VOSS); and (c) recounting everyday experiences related to pain.

### The virtual community of pain blogging: engaging a network of social support in the virtual environment

Virtual social support occurred across 100% of analyzed blogs, with bloggers and readers being involved in giving, seeking and receiving support through comment exchanges. Bloggers and readers played consoling and supportive roles in responses to blog posts about comments about personal experiences. Of the published comments, 16% provided consolation and included phrases such as “I’m sorry to hear” and “I completely understand”. In addition, it was not uncommon for readers to console bloggers and other readers. For example, in response to a post about a blogger's experience of migraines, one reader commented, “I’m so sorry to hear about your circumstances. I hope the light breaks through the clouds for you.”

While bloggers received support through comments such as “Keep it up and you will meet your goals!”, many hoped to provide support to others. Twenty-six bloggers responded regularly to comments and provided messages of encouragement and support to their readers: “Hang in there, one day at a time!” Furthermore, 31 of the 44 bloggers supported their readers by ending at least one blog post with encouraging, motivating, empowering and/or inspiring statements: “Let's not let the things we can't change keep us from dreaming of more [or] dreaming of tomorrow.”

Readers expressed gratitude and commended bloggers for their efforts in 10% of comments analyzed: “Thank you for continuing to blog. Even if it's sporadically. You make such a huge impact.” These comments were indicative of support experienced by participants in the online virtual community. According to content within the blog websites, five blogs were named Best Fibromyalgia Blog of the Year across 2014 and 2015. This award recognizes the efforts of multiple bloggers each year in educating, inspiring and empowering their readers, in addition to sharing an insight into life with fibromyalgia. As readers are required to nominate blogs for this award, this suggests that readers found value in reading personal blogs about pain and similar experiences. It also highlights an existing network of support in the online chronic pain blogging community.

Nineteen of 44 bloggers included in this analysis articulated how they found value in sharing their experiences online, even if they did not reach a large audience: “If even one person comes across this [searching] the internet in pain looking for anything, I hope this blog will give them some comfort and peace of mind.” Seventeen bloggers described a lack of guidance available during their own chronic pain journeys and the opportunity to help others on their journeys as motivations for starting a blog. One blogger posted:

I wish someone could have [saved] me the trouble of having to learn so much of it the hard way. While that didn't happen for me, that doesn't mean you have to be in the dark about all the craziness that comes with this illness.

Bloggers also used their online platforms to increase the public's awareness of their health conditions and the need for more research to be undertaken. Twenty-two of the 44 bloggers described the importance of publishing these types of blog posts in creating more inclusive and understanding communities: “I feel it is important to raise awareness of what people actually go through. These illnesses aren't really well understood in the general public.”

Eighteen of 44 bloggers documented their involvement in campaigns and events to raise awareness for conditions causing chronic pain in at least one post in their blogs. For example, one blogger posted: “For the next 48 hours, I am live-tweeting my #ChronicLife, and giving a portrayal of one chronic patient's reality.” Participation in these events, often using other social media platforms, encouraged other bloggers to do the same: “She was going to be live tweeting 48 hours in her life … I was inspired, and decided I would join her.” Documenting the participation of other bloggers in events created an introduction for readers to discover new blogs leading to more possibilities of engaging in social support exchanges.

### The Virtual Online Support Sequence (VOSS): establishing social support through blogging

A sequence of steps through which social support was garnered was identified within the comment exchanges from the virtual community of blogging. This pattern was identified in 32% of blog posts analyzed with 38 of 44 bloggers engaging in this social support mechanism. When readers were able to relate to the experiences described by bloggers, they would comment on blog posts with their similar experiences resulting in provision of social support. A sense of relatedness to parallel experiences pertained to both negative experiences with family and friends and poor interactions with health professionals. Connecting with these shared experiences appeared to provide comfort and reduce feelings of isolation among bloggers and blog readers.

This pattern has been identified in the current study, and accordingly named as the Virtual Online Support Sequence (VOSS); it involves blogging, relating, exchanging support and benefiting. Blogging involved writing a blog post and making it publically available. Relating involved commenting on the blog to share a similar experience. Encouraging, supportive, or empathetic statements were provided by bloggers, or other readers in the exchanging support step. In the benefiting step, the reader expresses experiencing a sense of comfort, reduced isolation or other psychosocial benefit by engaging in the comment exchange associated with the blog post. Table 2 contains three examples of this process. Most often, all of these steps were followed in the order outlined. However, in some instances, some steps were omitted. For example, in some exchanges blogging, exchanging support and benefiting occurred without the relating step.

**Table 2.** Examples of the Virtual Online Support Sequence (VOSS).

VOSS step	1. Blogging	2. Relating	3. Exchanging support	4. Benefiting
Description	Blogger describes their experiences in blog post	Reader comments on blog post with a similar experience	Blogger or another reader responds to reader's comments with encouragement, support or empathy	Reader expresses experiencing a sense of comfort, reduced isolation or other psychosocial benefit
Example 1	“Friends do come and go, more so when people are ill and more isolated from friendship groups … It can be a lonely process.”	“I am really struggling with my health this year and reading your blog has been really meaningful to me.”	“I am sorry to hear you are struggling with your health but I’m glad my blog and my experiences of ill health can give you something to relate too.”	“I can definitely relate to some of what you write about and it makes me feel less alone.”
Example 2	“Bring up an illness … and all of a sudden you are a faking complainer”	“So very many people have told me I’m a faker, or suck it up you're young. They don't get it.”	“Sorry that you have to go through this.”	“Thank you so so much for writing this. I teared up reading it because it hits home.”
Example 3	“We told [insurance] of my numerous bad reactions to other DMARDS … of my need for pain management, my inability to hold down a normal job … This wasn't enough apparently. DENIED.”	“After Enbrel and Orencia stopped working, I tried Xeljanz. I got my life back. We had to fight a few times to get insurance to cover it.”	“I am so glad to hear it is working for you.”	“Thank you for your message of HOPE!”

### Recounting everyday experiences related to pain

Experiences from everyday life were recounted and shared in 71% of published blog posts. Sharing individual experiences related to pain allowed blog users to initiate comment exchanges for virtual social support. In particular, the VOSS was evident when bloggers described their immediate social environments, the health service environment and their financial circumstances. Often, a single blog post discussed one or more of these environments and the discussions of them by blog users are described below.

Experiences from bloggers’ immediate social environment, usually of interactions with family and friends, were discussed in 26% of blog posts. Some bloggers expressed gratitude for having supportive family and friends who understood, to the best of their ability, their experiences of pain: “I am the luckiest girl in the world to be surrounded by the most incredibly supportive friends. The level of tolerance and understanding and accepting without understanding is incredible.” However, bloggers predominantly posted stories regarding a lack of understanding about their invisible illnesses from family and friends, stating that this often led to them masking their experiences of pain. For example, one blogger posted, “Often people close to us do not know what to say … or they say or do the “wrong” things … we may feel we have to put a mask on and fake feeling okay.”

Some bloggers and readers described experiencing a loss of support from family and friends due to a lack of understanding. One blogger recalled, “My husband left me a few months after my diagnosis with Fibro”, while another said, “The few relationships I had left eroded into a toxic sludge of confusion and misunderstanding.”

The VOSS identified earlier came into play when bloggers described negative experiences from their immediate social environment. This is evident in Table 2, Example 2, which illustrates how the VOSS can provide a supportive online environment for bloggers and readers to share these experiences of their immediate social environment.

Blog posts and discussions about health service environments included experiences with health professionals and the public health system and occurred in 16% of blog posts included in analysis. Bloggers frequently discussed their challenges in finding a supportive doctor to help with pain management, often describing their need to go “doctor shopping”: “He was already such a bad doctor I was looking for a new doctor due to his rudeness.” Many bloggers attributed negative experiences during their appointments with medical doctors to lack of recognition of their pain experiences and lack of listening to their needs: “I had a ridiculous appointment yesterday. My specialist asked me what was new, I went through my issues in order of importance. He ignored everything I said.”

While 27 bloggers discussed their negative experiences with health professionals, 3 bloggers spoke of positive experiences. These bloggers credited their experiences to being comfortable with and confident in their doctors. One blogger posted, “Last week I had an appointment at the hospital to see a specialist to do with my Joint Hypermobility syndrome … It was extremely a positive appointment that has left me feeling very optimistic.”

Bloggers and readers were also dissatisfied when miscommunications between health professionals occurred leading to them receiving conflicting information from different health professionals. For example, one blogger posted:

On Tuesday I attended a physiotherapy appointment, I was under the impression that this was to be the first of at least weekly appointments …. So you can imagine my total shock on Tuesday when my [physiotherapist] told me that my Neurologist had advised her to discharge me.

Dissatisfaction was also expressed when they felt the public health and insurance system environments were restricting their access to treatment options. Bloggers predominantly described their frustrations at these systems and the resultant difficulties in seeking healthcare:

The physical [therapists] can only focus on one specific diagnosis or part of the body at a time. This means that your physical therapist has to send you back to your primary care doctor, to get a new prescription and a new insurance authorization, just to be able to answer a question about a different part of the body than what they were treating.

Again, the VOSS occurred when health service environments were discussed on blogs. However, unlike previous examples, bloggers benefited from this support exchange when their readers consoled and comforted them as exemplified in Example 3 in Table 2.

Discussion of financial environments centered on the difficulties in receiving financial assistance and the expense of pain management options and appeared in 4% of analyzed blog posts. Some bloggers detailed their difficulties in receiving financial assistance from their healthcare systems in the hope that their experiences would provide their readers with advice on how to make successful applications. This often prompted readers to comment on their similar difficulties with accessing financial aid: “I too live with 24/7 chronic pain and have been fighting for disability for 5 years and been denied twice.” In relation to the VOSS, blogging and relating were evident during discussions about obtaining financial assistance.

Difficulties receiving financial assistance were discussed in five blogs. Bloggers and readers attributed their declined financial assistance applications due to assessors not being impartial and disregarding their experiences of pain. One blogger described their frustration and wrote, “Now got a letter refusing PIP [Personal Independence Payment], looking through the report nothing we said seemed to have been taken into account, nothing we told the assessor seems to have been recorded.”

Seven bloggers expressed their frustrations at the expensive costs of treatment options for managing pain, which were often compounded by their inability to work. One blogger posted, “Many people with chronic illness either can't work, or they have to reduce the amount of work they do … people with an illness or disability face barriers in employment and they face huge healthcare costs.” As a result, bloggers and readers reported having to save money for medical expenses: “It is good to save money … I do this because of the astronomical amount of money we pay for my health insurance and medicals costs.”

Blog posts describing financial experiences again initiated the VOSS among blog users. Comment exchanges allowed blog readers to benefit from bloggers sharing their personal experiences and advice about successful financial assistance applications.

## Discussion

This research aimed to identify the means by which blog users who have chronic pain are able to access virtual social support by connecting and relating to experiences published online by bloggers. The findings indicate that people with chronic pain do this by using a 4-step sequence involving blogging, relating, exchanging support and benefiting. In this research, this sequence has been named the VOSS.

The current body of research supports the use of online social platforms in establishing relationships for virtual social support.^13,18,19,21,24–29^ Specifically, documenting and communicating experiences of chronic pain through blogging has been recognized to foster an environment that allows the growth of online relationships.^7^ There is a current understanding that relationships between bloggers and readers can be established through comment exchanges.^13,18–20^ Blogging allows bloggers to document and share their experiences of what it means to live with chronic pain and create opportunities for readers to relate to the experiences published.^3,21^ Blog readers who comment on blog posts can engage in a back-and-forth online process of providing and receiving emotional support that mirrors the mechanisms of face-to-face support.^13,21^ Such exchanges afford blog users with opportunities to comment in order to give, seek and receive social support more readily from blogs.^15,21^ The VOSS identified in this research captures the cyclical nature of face-to-face interactions, in that support occurs through a process of blogging, relating, exchanging support and benefiting. This suggests that online relationships can be formed between blog users who share similar experiences of chronic pain. When bloggers and readers engage in comment exchanges relating to these shared circumstances, virtual social support can be experienced. Therefore, findings from the present study contribute to the current understanding of how blogging can garner social support by identifying a mechanism behind the formation of supportive online relationships. Future research might consider examining blogs over time to analyze the ongoing interactions between blog users, and not just the interactions specific to each blog post independently. This would provide further understanding of the ways deeper, long-term relationships might be built between blog users for social support.

Social support is a multicomponent and multidimensional construct and can appear in the forms of informational, emotional and instrumental support.^36,37^ Receiving social support from family and friends has been posited to safeguard against the psychological effects of chronic pain as it can help build resilience to cope with stressful events and experiences.^7,38,39^ Social support has been found to influence chronic pain severity as sources of support can encourage the use of coping strategies.^37^ Previous research also suggests that a combination of emotional support from family or significant others with informational support from physicians can assist the acceptance process for people with chronic pain.^16^ Furthermore, people with chronic pain are also more likely to participate in daily, social, vocational and leisure activities when they are encouraged and emotionally supported by others.^13,20–23^ However, social support can often be limited for people with chronic pain due to the persistent physical, psychological and invisible nature of the pain.^4,5,6^ While this research does not identify the outcomes of virtual social support, it has identified the process through which social support can occur via social media, in particular through blogging.

The current study illustrates how people with chronic pain can interact with others through the VOSS in order to gain informational and emotional social support, particularly regarding the challenges they face in offline environments. Blog readers were able to seek informational support from bloggers by reading documented experiences or by commenting questions. The back and forth nature of the VOSS facilitated both bloggers and readers to seek and provide emotional social support through expressions of encouragement and empathy. Thus blogging, or reading and commenting on blogs, can provide an alternative or supplementary source of social support for people with chronic pain when their support networks are limited. Blogging, or engaging in an online community, could be added to health professionals' toolbox of pain management strategies that they share with their clients. Through knowledge of the virtual social support mechanism, individuals can be encouraged to actively engage with bloggers through commenting and leaving messages on blog posts they relate to.

In this research, the VOSS was identified in topics that discussed experiences of the immediate social, health service and financial environments. Therefore, clinicians can provide real-life examples of the VOSS to encourage their clients to go online to obtain support. Understanding the VOSS can give individuals a reason to share their experiences; as indicated by this mechanism, sharing experiences creates opportunities for others to experience relatedness, which can lead to virtual social support. This is important, because many people with chronic pain lack the opportunity to honestly communicate about their lived experience, not because family and friends do not care, but because they do not understand or they do not listen.^4,5^ Current findings suggest that encouraging people experiencing chronic pain to seek support online can enable them to receive, give and seek the support they require within the comfort of home. Future research investigating the impacts of virtual support received through blogging on everyday participation can strengthen the clinical utility of the VOSS.

These findings offer a first step in understanding the process through which social support occurs online for people with chronic pain. The VOSS might be transferable to other social media platforms; however, further research is needed to investigate similarities and differences in the VOSS across a range of social media platforms such as Facebook, Twitter, Instagram, and Snapchat. Further research is also needed to explore the relevance of the VOSS across different cultures and how differences in social media use can impact on accessing virtual social support. If the VOSS is transferable to other platforms, health professionals could support people with chronic pain to use the VOSS in order to strengthen their social support regardless of their preferred social media platform.

For a relationship to be formed virtually, engagement in forms of commenting and replying is required of both bloggers and readers. However, a lack of physical proximity and face-to-face contact can act as barriers to forming genuine supportive online relationships.^40,41^ Furthermore, the capacity to provide, receive or seek immediate online support can be limited by readers who do not actively engage in comment exchanges.^29^ This can create challenges as comments, questions and messages may be left unanswered. For people with chronic pain who are already at risk of social isolation,^40,42^ the potential for further isolation from online platforms is of concern as the later stages of the VOSS, exchanging support and benefiting, may not occur.

Despite online platforms providing opportunities for virtual social support, negative online experiences may deter blog users from publishing or commenting on blog posts. In some instances, blog users may stop engaging in the online community altogether.^43^ This lack of active engagement can compromise the ability to seek social support online through blogging. Negative experiences may arise from misinterpretations of comments posted and can influence the quality of comment exchanges. Additionally, the ability to remain anonymous when commenting on blog posts has been identified to facilitate cybervictimization,^43^ which can have negative influences on the mental health and wellbeing of blog users.^43–45^ This present study does not report on the implications of negative experiences of blogging on the VOSS; however, further research is needed to explore the influences of such negative online experiences on the availability and sustainability of virtual social support established through blogging.

While the risk of further isolation through negative experiences may exist, blogging provides people with chronic pain new opportunities to form relationships. Through the VOSS, social support can be garnered from blog users who share a mutual understanding of pain. This can decrease feelings of isolation, encourage confidence in pain management and promote overall wellbeing.^21^

### Limitations

The VOSS has been identified from blog posts authored by bloggers experiencing chronic pain. While chronic pain features as a symptom of many different health conditions, the generalizability of these findings cannot be assumed. Future research might determine whether this mechanism of support may be applicable to particular diagnoses or causes of chronic pain, other health conditions and/or other non-health related topics. As this research investigated experiences of blogging, future research might also investigate if the VOSS occurs in other virtual environments across social media.

A sample of blog posts was included in this study; specifically only the first blog post of each month, over a 24-month period was included for analysis. While this reduced the overall number of blog posts analyzed, there was still sufficient and meaningful data to provide an overview of the progression of experiences of pain. Moreover, limiting the amount of data analyzed allowed for manual coding and theming, which resulted in retention of higher-level meaning expressed in the blogs throughout the analysis process. Future research might triangulate data collected from blogs with data from interviews with blog users to confirm and provide further insights into the experiences of chronic pain blog users.^30^

This study did not analyze images and photographs used in the blogs. Adding pictures (original or re-blogged from other web/blog locations) to supplement blog posts or communicate illness experiences was a feature across all blog-hosting platforms. Research has recognized that relatable and memorable pictures generate feelings of identity and can initiate online conversations.^18,19^ As only original written text from blog posts was analyzed in this study, understanding of blogging using pictures might provide additional insights into the VOSS or other aspects of social support and blogging by people with chronic pain. Future research might investigate the role of pictures (original or re-blogged) in posts in the VOSS for blog users seeking virtual social support.

## Conclusions

This research aimed to examine the experiences and interactions between people with chronic pain who blog about their experiences and other people who live with chronic pain. Study findings extend the current understanding about online blogging as an avenue of social support by identifying the use of a VOSS by bloggers and blog readers which enables the establishment of a virtual “community”. It provides a conceptual understanding of how bloggers and readers who share experiences of chronic pain can establish relationships. Through sharing experiences of different offline environments related to pain, blog users are able to engage in a sequence of steps leading to experiences of virtual social support. This mechanism by which virtual social support can be established provides new insights into the way in which health professionals can provide support for people with chronic pain in accessing additional support through the online blogging environment. Further research into the VOSS could be carried out to determine if it occurs across other blogs and other virtual environments focusing on a wider variety of health conditions.

## Supplemental Material

Supplementary Table 1 -Supplemental material for Seeking virtual social support through blogging: A content analysis of published blog posts written by people with chronic pain Click here for additional data file. Supplemental material, Supplementary Table 1 for Seeking virtual social support through blogging: A content analysis of published blog posts written by people with chronic pain by Samuel Tsai, Emma Crawford and Jenny Strong in Digital Health
