# Supplementary material for: Seeking virtual social support through blogging: A content analysis of published blog posts written by people with chronic pain
Source: Digit Health. 2018 May 16;4:2055207618772669. doi: 10.1177/2055207618772669 (PMC6016559; doi:10.1177/2055207618772669)
Supplement: Supplementary Table 1 -Supplemental material for Seeking virtual social support through blogging: A content analysis of published blog posts written by people with chronic pain [file Supplementary_Table_1.pdf]

Supplementary Table 1. Themes, categories and codes identified using qualitative phenomenological thematic analysis.

<table>
<tr><td colspan="4"><u>First Order Theme</u>: Social support in the online blogging environment</td></tr>
<tr><td><u>Second Order Themes</u></td><td><u>Third Order Themes</u></td><td><u>Fourth Order Themes</u></td><td><u>Codes</u></td></tr>
<tr><td rowspan="13">Virtual environment</td><td rowspan="5">Life as a blog author</td><td rowspan="2">Motivations for blogging</td><td>Lack of guidance</td></tr>
<tr><td>Addressing lack of understanding through lived experiences</td></tr>
<tr><td>Product review opportunities</td><td rowspan="3"></td></tr>
<tr><td>Difficulty writing blog posts</td></tr>
<tr><td>Raising awareness</td></tr>
<tr><td rowspan="8">Virtual social support</td><td>Recognizing author’s efforts</td><td rowspan="8"></td></tr>
<tr><td>Consoling others/author</td></tr>
<tr><td>Sense of relatedness</td></tr>
<tr><td>Encouraging engagement</td></tr>
<tr><td>Motivating others</td></tr>
<tr><td>Forming connections</td></tr>
<tr><td>Empowering others</td></tr>
<tr><td>Inspiring others</td></tr>
<tr><td rowspan="2">Financial limitations</td><td>Expensive costs of pain</td><td colspan="2" rowspan="2"></td></tr>
<tr><td>Difficulty receiving financial assistance</td></tr>
<tr><td rowspan="2">Interactions with health professionals</td><td>Lack of recognition of pain</td><td colspan="2" rowspan="2"></td></tr>
<tr><td>Miscommunication between health professionals</td></tr>
</table>

<table>
<tr><td></td><td>Limited help from insurance</td><td colspan="2"></td></tr>
<tr><td rowspan="13">Social environment</td><td rowspan="8">Lack of understanding from others not in pain (family and friends)</td><td rowspan="4">Difficulty communicating with others about pain</td><td>Masking pain from others</td></tr>
<tr><td>Perceived ideas of pain</td></tr>
<tr><td>Lack of knowledge about pain</td></tr>
<tr><td>Insensitivity from others</td></tr>
<tr><td>Concept of invisible illness</td><td rowspan="4"></td></tr>
<tr><td>Judgment from others not in pain</td></tr>
<tr><td>Lack of inclusive environments</td></tr>
<tr><td>Loss and/or lack of support systems</td></tr>
<tr><td>Support from family</td><td colspan="2" rowspan="5"></td></tr>
<tr><td>Support from friends</td></tr>
<tr><td>Appreciating support from others</td></tr>
<tr><td>Importance of respecting each other</td></tr>
<tr><td>Negative social experiences</td></tr>
<tr><td colspan="4"><u>First Order Theme</u>: Experiences of life with pain</td></tr>
<tr><td><u>Second Order Themes</u></td><td><u>Third Order Themes</u></td><td colspan="2"><u>Fourth Order Themes</u></td></tr>
<tr><td rowspan="4">Lived experiences of pain</td><td>Comparing self to others</td><td colspan="2" rowspan="4"></td></tr>
<tr><td>Diagnosis difficulties</td></tr>
<tr><td>Feelings of frustration</td></tr>
<tr><td>Lack of guidance</td></tr>
</table>

	Difficulties living with pain	
	Changes to life due to pain	
	Impacts of pain on others	
	New pain tolerance levels	
	Unexpected experiences of pain	
	Environmental influences of pain	
	Exacerbation of pain and symptoms	
	Unpredictable nature of pain	
	Triggers of pain	
	Progression of pain	
	**Emotional impacts of pain**	Loss of hope
		Sense of isolation
		Expressing guilt
		Sense of helplessness
		Fear of unknown
	Mental health impacts of pain	
	Lack of acceptance of pain into life	
	Loss of control in life	
**Coping with pain**	Coping strategies (adaptive and maladaptive)	
	Learning to cope with pain	
	Difficulty coping with pain	
**Managing pain**	Difficulty finding effective	

	management options	
	Personalized pain management	
	Frequent hospital visits	
	Managing multiple conditions	
	Listening to body (knowing limits)	
	Self-identified management strategies	
	Minimizing the impacts of pain	
	Dietary influences on pain	
	Alternative medicine	
	Managing pain through exercise	
	Self-medicating pain	
	**Medication**	Withdrawal effects of medication
		Medication dependence
		Difficulty accessing medication
		Difficulty finding effective medication
		Medication abuse
		Benefits of medication
		Medication cessation
		Stigma surrounding medication use
		Side effects of medication
**Surviving pain by overcoming challenges**	Resilience	
	Strength and determination	

	Taking control of life	
	Advocating for self	
**Changing outlook**	Acceptance of pain	
	Hope for the future	
	Stepping out of comfort zone	
	Regaining confidence	
	Identity beyond pain	
	Staying positive	
	Personal growth over time	
	Seeking help from others	
	Positive body image	
**Participation in everyday life**	Engaging in meaningful activities and roles despite pain	
	Achieving despite pain	
	Finding motivation to engage	
	Using assistive technology	
	Enjoying life/chasing dreams	

First Order Theme: **Sharing information with readers**		
Second Order Themes	Third Order Themes	Fourth Order Themes
Sharing resources		
Research updates		
Pain education		

This paper focused on the first, first order theme presented in the table: social support in the online blogging environment. Similar codes have been group together into 18 different categories, these categories are bolded in the table. These 18 categories were then ordered into four different levels of themes.
